# Expected and observed in‐hospital mortality in heart failure patients before and during the COVID‐19 pandemic: Introduction of the machine learning‐based standardized mortality ratio at Helios hospitals

**DOI:** 10.1002/clc.23762

**Published:** 2021-12-23

**Authors:** Sebastian König, Vincent Pellissier, Johannes Leiner, Sven Hohenstein, Laura Ueberham, Andreas Meier‐Hellmann, Ralf Kuhlen, Gerhard Hindricks, Andreas Bollmann, Helios hospitals

**Affiliations:** ^1^ Department of Electrophysiology Heart Center Leipzig at University of Leipzig Leipzig Germany; ^2^ Leipzig Heart Institute Leipzig Heart Digital Leipzig Germany; ^3^ Helios Hospitals Berlin Germany; ^4^ Helios Health Berlin Germany

**Keywords:** administrative data, COVID‐19 pandemic, heart failure, machine learning, mortality prediction

## Abstract

**Background:**

Reduced hospital admission rates for heart failure (HF) and evidence of increased in‐hospital mortality were reported during the COVID‐19 pandemic. The aim of this study was to apply a machine learning (ML)‐based mortality prediction model to examine whether the latter is attributable to differing case mixes and exceeds expected mortality rates.

**Methods and Results:**

Inpatient cases with a primary discharge diagnosis of HF non‐electively admitted to 86 German Helios hospitals between 01/01/2016 and 08/31/2020 were identified. Patients with proven or suspected SARS‐CoV‐2 infection were excluded. ML‐based models were developed, tuned, and tested using cases of 2016–2018 (*n* = 64,440; randomly split 75%/25%). Extreme gradient boosting showed the best model performance indicated by a receiver operating characteristic area under the curve of 0.882 (95% confidence interval [CI]: 0.872–0.893). The model was applied on data sets of 2019 and 2020 (*n* = 28,556 cases) and the hospital standardized mortality ratio (HSMR) was computed as the observed to expected death ratio. Observed mortality rates were 5.84% (2019) and 6.21% (2020), HSMRs based on an individual case‐based mortality probability were 100.0 (95% CI: 93.3–107.2; *p* = 1.000) for 2019 and 99.3 (95% CI: 92.5–106.4; *p* = .850) for 2020. Within subgroups of age or hospital volume, there were no significant differences between observed and expected deaths. When stratified for pandemic phases, no excess death during the COVID‐19 pandemic was observed.

**Conclusion:**

Applying an ML algorithm to calculate expected inpatient mortality based on administrative data, there was no excess death above expected event rates in HF patients during the COVID‐19 pandemic.

**Abbreviations**
AUC: area under the curve
AUPRC: area under the precision‐recall curve
CI: confidence interval
HF: heart failure
HSMR: hospital standardized mortality ratio
ICD‐10: International Statistical Classification of Diseases and Related Health Problems
ML: machine learning
NYHA: New York Heart Association
ROC: receiver operating characteristic

## 1 INTRODUCTION

During the early phase of the ongoing COVID‐19 pandemic, numbers of heart failure (HF)‐related hospital admissions were significantly decreased.^1^, ^2^, ^3^, ^4^, ^5^, ^6^ This was accompanied by an increase in case severity with regard to New York Heart Association (NYHA) class and higher in‐hospital mortality rates.^5^, ^7^, ^8^, ^9^ It is unclear, whether the inferior outcome had to be attributed only to differing patient profiles or to additional factors like changes in HF patient care during the pandemic. Risk‐adjusted mortality prediction would allow standardized modeling with regard to time intervals and regional differences of endpoints like in‐hospital mortality. We previously introduced different machine learning (ML)‐based algorithms for the calculation of expected mortality rates on a populational level in a large German HF cohort that only implemented widely accessible administrative data.^10^ ML models outperformed logistic regression analysis according to values of the area under the curve (AUC) with the extreme gradient boosting model showing the best performance metrices. The aim of the present analysis was to apply this model to data from 2019 to 2020 of the same nationwide, real‐world data set and compare expected and observed mortality rates with respect to pandemic phases overall and in specific subgroups.

## 2 METHODS

### 2.1 Data source

Administrative data of 86 German Helios hospitals was retrospectively analyzed. Patient cases with full inpatient treatment between January 1, 2016 to August 31, 2020 and the main discharge diagnosis of HF defined in accordance to prior publications were identified.^5^, ^10^ Hospital admission and discharge were categorized within administrative data and only cases with both urgent (nonelective) admission and hospital discharge type other than hospital transfer were further analyzed. Discharge diagnoses were encoded using the International Statistical Classification of Diseases and Related Health Problems (ICD‐10‐GM [German Modification]). Comorbidities were identified from encoded secondary diagnoses at hospital discharge according to the Elixhauser comorbidity score.^11^, ^12^ All patients with an encoded SARS‐CoV‐2 infection (U07.1, U07.2!) were excluded. Cases with missing information for NYHA classes (*n* = 7280) were discarded due to an adequate calibration of ML models. Detailed information regarding used ICD‐codes is provided in the Supporting Information Material (Tables S1 and S2). We computed the number of laboratory‐proven SARS‐CoV‐2 infections per 100,000 inhabitants within a federal state using data from the Robert‐Koch‐Institute and the Federal Bureau of Statistics (Germany) with tertiles defining areas with low (<152), intermediate (152–297), and high COVID‐19 case volume (>297).^13^ Hospitals were categorized with respect to the number of yearly HF admissions between 2016 and 2018 and expressed as tertiles with low (<149), intermediate (149–368), and high (>368) hospital case volume. Curves of daily admissions for 2019 and 2020 were fitted using locally estimated scatterplot smoothing with a degree of smoothing of *α* = .25 and with corresponding 95% confidence intervals (CIs). Based on nonoverlapping CIs for daily hospital admissions defining the beginning and end of the deficit period with fewer HF‐related hospitalizations in 2020, this deficit period lasted from 03/12/2020 to 04/14/2020 (corresponding phase in 2019 with respect to changing weekdays: 03/13/2019–04/15/2019). The time interval before and after the nonoverlapping CIs will be considered the prepandemic period (2020: 01/01/2020–03/11/2020, corresponding phase in 2019: 01/01/2019–03/12/2019) and the resumption period (2020: 04/15/2020–08/18/2020, corresponding phase in 2019: 04/16/2019–08/19/2019) as illustrated in Figure S1. Patients' data were stored in a pseudonymized form and data use was approved by the local ethics committee (AZ490/20‐ek) and the Helios Kliniken GmbH data protection authority. Considering the retrospective analysis of double‐pseudonymized administrative clinical routine data, individual informed consent was not obtained.

### 2.2 Model development/testing

All analyzes were performed within the R environment for statistical computing (version 3.6.1, 64‐bit build).^14^ Data from 2016 to 2018 (*n* = 59,125 cases from 69 German Helios hospitals, 69.8% aged ≥75 years, 51.9% female) was split into 75%/25% portions used for model development and testing, stratified for in‐hospital mortality. Baseline characteristics were well balanced between the data set parts as can be seen in Table S3. Random forest, gradient boosting machine, single‐layer neural network, and extreme gradient boosting were the machine ML‐based models being investigated and compared to logistic regression. Variable selection and scaling as well as model tuning were performed as described previously.^10^ Final model adaptations including a recalibration of approximated probabilities using a generalized additive model and a reclassification of thresholds based on receiver operating characteristic (ROC) curves and F1 statistics have been carried out according to our previous work.^10^ Using the probabilities predicted within the test data and the optimal threshold, the predictive abilities of the algorithm were assessed by the ROC AUC, the precision‐recall curve, the area under the precision‐recall curve (AUPRC), calibration‐in‐the‐large (overall expected and observed mortality rate), weak calibration (intercept and slope of the calibration curve), calibration plots, F1 statistic and confusion matrices.^15^ Pooled in‐hospital mortality rate within the development data set was 6.20%. Extreme gradient boosting was the most reliable ML model with respect to the highest ROC AUC (0.882, 95% CI: 0.872–0.893) and AUPRC (0.477, 95% CI: 0.445–0.509) in the test data set when implementing the Elixhauser comorbidities as individual variables.^10^ Calibration of the model showed adequate accordance of predicted and observed events within the test data set, the Brier score (uncalibrated) was 0.043 without an improvement after recalibrating probabilities. Therefore, the extreme gradient boosting model was utilized for further model application in this study. Specific variable importance values for baseline variables and Elixhauser comorbidities are listed in the Supporting Information Material (Table S4).

### 2.3 Calculation of expected mortality rates

The model was used to calculate the expected number of deaths in 2019 and 2020 (admissions limited to August 31st) as the sum of individual in‐hospital mortality probabilities. The hospital standardized mortality ratio (HSMR) was computed as the ratio between observed and predicted deaths. Its 95% CI was calculated using Byar's approximation. HSMRs within years were compared using the Spearman rank correlation.

## 3 RESULTS

**Figure 1:**
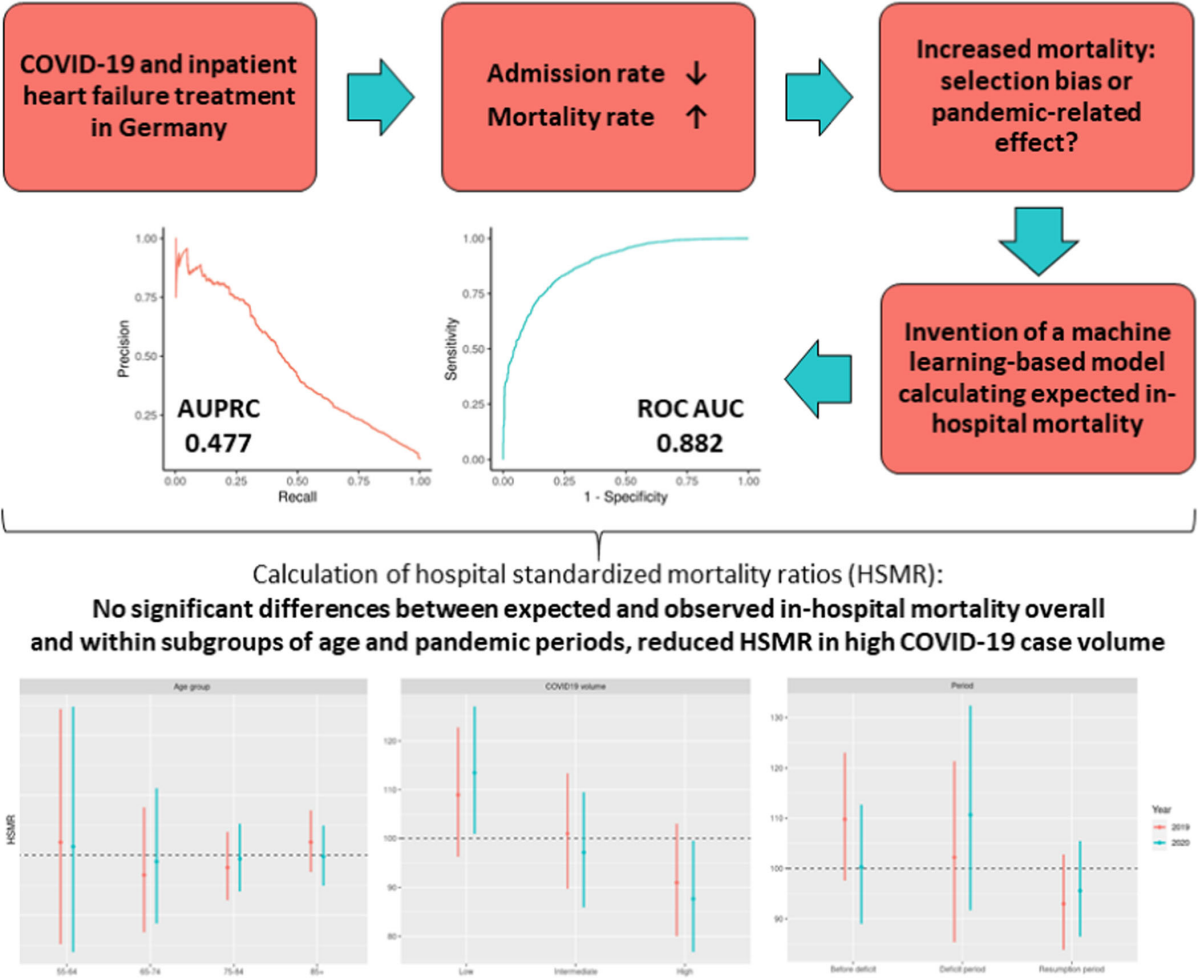
Hospital standardized mortality ratios within several subgroups in 2019 and 2020

In this retrospective cross‐sectional analysis, 26,591 patient cases from 2019 to 2020 were analyzed and in‐hospital mortality was predicted using the extreme gradient boosting machine model to compare differences between predicted and observed mortality rates throughout the years. Comparing baseline characteristics, patients were older, had a different composition of comorbidities, and had a shorter length of stay in 2019 and 2020 compared to the data set of 2016–2018. Cases from the model development cohort also were more symptomatic as reflected by a higher percentage of patients classified as NYHA class IV, whereas NYHA class III was overrepresented in 2019 and 2020. Baseline characteristics are listed in Table 1. Overall, observed mortality rates were 5.89% in 2019% and 6.23% in 2020, corresponding HSMRs based on the individual case‐based mortality probability were 100.0 (95% CI: 93.3–107.2; *p* = 1.000) for 2019 and 99.3 (95% CI: 92.5–106.4; *p* = .850) for 2020. HSMRs were further studied in subgroups stratified by age, predefined pandemic phases in2020 and corresponding time intervals in 2019, hospital volume and COVID‐19 case volume. Within the subgroups of age, there were no significant differences between predicted and observed in‐hospital mortality rates for both 2019 and 2020. When stratified for the different pandemic phases, HSMRs in 2019 and 2020 were 109.8 (95% CI: 97.6–123.0; *p* = .118) and 100.3 (95% CI: 89.0–112.7; *p* = .971) for the prepandemic period, 102.2 (95% CI: 85.4–121.4; *p* = .828) and 107.5 (95% CI: 91.7–132.4; *p* = .291) for the deficit period as well as 93.0 (95% CI: 83.9–102.8; *p* = .159) and 95.6 (95% CI: 86.5–105.4; *p* = .382) for the resumption period, respectively. In areas with high COVID‐19 case volume, observed death rates were lower than the predicted ones for both 2019 (HSMR: 91.0; 95% CI: 80.0–103.1; *p* = .142) and 2020 (HSMR: 87.6; 95% CI: 76.8–99.5; *p* = .042) with only the latter meeting the criteria of statistical significance. HSMRs were not different from 100 in intermediate COVID‐19 case volume regions in both years and in low COVID‐19 case volume regions in 2019, but a difference between expected and observed mortality with an HSMR of 113.5 (95% CI: 100.9–127.1; *p* = .034) was calculated for the year 2020 in areas with low COVID‐19 case volume. No differences with respect to HSMRs were observed for the different groups of hospital volume. HSMRs within subgroups are presented in detail in Table 2 and illustrated in Figure 1.

**Table 1:** Baseline characteristics comparing datasets used for model development with data of 2019 and 2020

Variable	Model development	2019	2020	*p*
*N*	59 125	13 690	12 901	
Age (years)				
<65	12.6% (7459/59 125)	11.5% (1568/13 690)	11.0% (1424/12 901)	<.001
65–74	17.6% (10 377/59 125)	17.5% (2390/13 690)	16.3% (2103/12 901)	.003
>75	69.8% (41 289/59 125)	71.1% (9732/13 690)	72.7% (9374/12 901)	<.001
Length of stay (days)				
<5	37.2% (21 981/59 125)	40.0% (5478/13 690)	40.9% (5271/12 901)	<.001
5–9	32.8% (19 392/59 125)	32.0% (4382/13 690)	33.0% (4252/12 901)	.163
>9	30.0% (17 752/59 125)	28.0% (3830/13 690)	26.2% (3378/12 901)	<.001
Intensive care Length of stay (days)				
0	80.3% (47 485/59 125)	84.0% (11 500/13 690)	82.5% (10 644/12 901)	<.001
>0	19.7% (11 640/59 125)	16.0% (2190/13 690)	17.5% (2257/12 901)	<.001
Gender				
Female	51.9% (30 689/59 125)	51.0% (6987/13 690)	51.1% (6588/12 901)	.069
Male	48.1% (28 436/59 125)	49.0% (6703/13 690)	48.9% (6313/12 901)	.069
NYHA class				
NYHA II	8.9% (5289/59 125)	9.2% (1265/13 690)	7.7% (997/12 901)	<.001
NYHA III	42.0% (24842/59 125)	45.3% (6200/13 690)	46.4% (5986/12 901)	<.001
NYHA IV	47.4% (28027/59 125)	44.3% (6061/13 690)	44.9% (5798/12 901)	<.001
Elixhauser comorbidities				
Cardiac arrhythmias	62.4% (36 921/59 125)	63.0% (8630/13 690)	64.2% (8288/12 901)	.001
Valvular disease	37.7% (22 269/59 125)	39.9% (5468/13 690)	38.5% (4967/12901)	<.001
Pulmonary circulation disorders	19.2% (11 357/59 125)	18.9% (2590/13 690)	17.3% (2235/12 901)	<.001
Peripheral vascular disorders	12.9% (7633/59 125)	11.9% (1635/13 690)	11.5% (1480/12 901)	<.001
Hypertension, uncomplicated	30.1% (17 800/59 125)	28.4% (3884/13 690)	30.3% (3914/12 901)	<.001
Hypertension, complicated	49.6% (29 343/59 125)	49.5% (6772/13 690)	48.1% (6208/12 901)	.008
Chronic pulmonary disease	19.5% (11 515/59 125)	18.9% (2584/13 690)	18.1% (2340/12 901)	.001
Diabetes, uncomplicated	18.0% (10 642/59 125)	17.6% (2405/13 690)	17.3% (2229/12 901)	.106
Diabetes, complicated	22.0% (13 028/59 125)	21.4% (2929/13 690)	22.3% (2882/12 901)	.149
Hypothyroidism	13.4% (7935/59 125)	14.2% (1944/13 690)	14.9% (1928/12 901)	<.001
Renal failure	63.0% (37 250/59 125)	62.5% (8554/13 690)	63.4% (8182/12 901)	.281
Obesity	23.3% (13 759/59 125)	23.3% (3186/13 690)	21.4% (2758/12 901)	<.001
Weight loss	6.0% (3548/59 125)	5.6% (771/13 690)	5.5% (704/12 901)	.027
Fluid and electrolyte disorders	31.3% (18 504/59 125)	30.6% (4188/13 690)	33.4% (4309/12 901)	<.001
Deficiency anemia	5.4% (3195/59 125)	5.5% (759/13 690)	6.7% (867/12 901)	<.001
Depression	5.3% (3121/59 125)	5.0% (680/13 690)	5.2% (673/12 901)	.336

**Table 2:** Predicted and observed mortality as well as HSMRs overall and within subgroups

	2019				2020			
Level	Observed	Predicted	HSMR (95% CI)	*p*	Observed	Predicted	HSMR (95% CI)	*p*
*N*	807	806.7	100.0 (93.3–107.2)	1.000	804	810.0	99.3 (92.5–106.4)	.850
Age group								
55–64	30	28.8	104.3 (70.3–148.8)	.868	27	26.3	102.8 (67.7–149.6)	.937
65–74	82	87.8	93.4 (74.3–115.9)	.581	77	78.7	97.8 (77.2–122.3)	.907
75–84	280	292.0	95.9 (85.0–107.8)	.503	304	307.8	98.8 (88.0–110.5)	.857
85+	407	390.2	104.3 (94.4–114.9)	.409	387	388.9	99.5 (89.8–109.9)	.951
Period								
Prepandemic period	296	269.6	109.8 (97.6–123.0)	.118	284	283.1	100.3 (89.0–112.7)	.971
Deficit period	130	127.2	102.2 (85.4–121.4)	.828	119	107.5	110.7 (91.7–132.4)	.291
Resumption period	381	409.8	93.0 (83.9–102.8)	.159	401	419.4	95.6 (86.5–105.4)	.382
Hospital volume								
Low	74	86.9	85.1 (66.8–106.9)	.177	68	79.7	85.3 (66.2–108.1)	.204
Intermediate	268	265.1	101.1 (89.4–114.0)	.873	279	298.1	93.6 (82.9–105.2)	.279
High	465	454.7	102.3 (93.2–112.0)	.640	457	432.1	105.8 (96.3–115.9)	.242
COVID19 volume								
High	248	272.5	91.0 (80.0–103.1)	.142	237	270.5	87.6 (76.8–99.5)	.042
Low	268	246.1	108.9 (96.3–122.8)	.174	298	262.7	113.5 (100.9–127.1)	.034
Intermediate	291	288.1	101.0 (89.7–113.3)	.879	269	276.9	97.1 (85.9–109.5)	.662

Abberviations: CI, confidence interval; HSMR, hospital standardized mortality ratio.

## 4 DISCUSSION

In this retrospective cross‐sectional analysis, we applied our previously introduced ML algorithm (gradient boosting machine) for the calculation of expected in‐hospital mortality rates on a populational level in a nationwide, multicenter cohort of HF patients containing administrative data before and throughout the COVID‐19 pandemic. Our model had high‐performance indices and was well‐calibrated. When comparing model‐derived expected with observed mortality for 2019 and 2020, we found regional differences of HSMR values but overall high accordance between calculated and true in‐hospital mortality rates. The relative increase of in‐hospital mortality in HF patient cohorts that has been previously observed during the COVID‐19 pandemic did not exceed the expected variation of death rates that were calculated by our model based on patients' baseline characteristics.^5^ There were no significant differences in overall HSMRs of 2019 and 2020. Consequently, the higher in‐hospital mortality rate in 2020 is likely attributable to the differing case mix with older patients suffering from a different composition of comorbidities. Increased in‐hospital mortality rates in 2020 compared to previous years were also reported by other groups, which were also related to differing baseline characteristics including a higher mean age and the presence of more comorbidities.^8^, ^16^ In contrast, other groups reported no or at least no significant differences in mortality rates in the same period.^1^, ^2^, ^4^ Considering the fact that no relevant variations of baseline characteristics were observed in the latter studies when comparing the different observational periods, this would underline the crucial role of the corresponding case mix of the cohorts of interest on in‐hospital mortality. This is therefore compatible with our findings of indifferent HSMRs.

Interestingly, observed mortality rates were even lower in areas with high COVID‐19 case numbers. There is no obvious explanation for this observation. Since a similar trend also was apparent in 2019, a fixed regional effect caused by unknown structural differences is likely to influence the results. For example, population density has been shown to impact in‐hospital outcomes in HF patients.^17^ An uneven distribution of cases discharged as hospital transfers between different areas could also contribute to this finding, as those cases were excluded from our analysis. This was done to avoid a biased in‐hospital death rate because no cross‐linking of patient cases between hospitals was possible due to data structure and data privacy. Moreover, an early discharge to prevent nosocomial infection and to keep capacities ready especially in areas being highly affected by incident SARS‐CoV‐2 infections could lead to the transfer of patients to the outpatient sector. Previous findings of a shortened length of stay during the pandemic are pointing in this direction.^2^, ^5^ Other studies reported higher rates of out‐of‐hospital cardiac arrests with an increased case‐fatality rate during the pandemic and overall excess mortality during the first half of 2020 in Germany compared to previous years.^18^, ^19^, ^20^, ^21^ Whether this is indicative of an actual shift of cardiovascular deaths from the hospital to the outpatient setting needs to be further studied.

The overall high concordance of expected and observed in‐hospital mortality rates within different age groups indicates a high reliability of the investigated model. There were only two comparable prediction models focusing on administrative data only, which reported lower AUC values (0.72–0.78).^22^, ^23^ A direct juxtaposition with our model is, however, hindered due to a different set of included variables. Our data set does not contain information on ethnicity, insurance data, used medication, and other variables being implemented into the mentioned models. Possible explanations for this better predictive performance, besides the algorithm itself, might be a higher event rate in our cohort affecting the model quality during the developmental stage. Furthermore, the cohort size of our training data set was significantly larger at least when compared to the study of Desai and colleagues.^22^ Both studies also examined whether the addition of data from electronic medical records would lead to an improvement of the predictive power and presented ambiguous results. Whereas one report propagated similar model discrimination when only using claims data, the other one showed better performance metrices when augmenting the administrative data set by laboratory results and imaging data.^22^, ^23^ Contrary, a previous study by Lagu et al. reported even better performance of administrative data‐based prediction models when compared to clinical prediction tools for HF patients.^24^ Other algorithms designed to forecast short‐ or long‐term mortality in acute as well as chronic HF showed a similar or lower discriminatory power even when including more sophisticated and disease‐specific variables.^25^, ^26^, ^27^, ^28^, ^29^, ^30^ Of course, it needs to be noticed that all of those prediction tools were developed to predict an individual risk and not all of them used in‐hospital death as the endpoint of interest. That being said, the juxtaposition of performance metrices can only serve to get an impression of the quality of our model instead of directly comparing it to the ones mentioned above. If confirmed as a reliable algorithm after external validation, our model also offers far‐reaching possibilities beyond analyzes linked to the COVID‐19 pandemic. This includes both the standardized comparison of HF‐related mortality in quality management programs with regard to temporal and regional differences of medical treatments as well as an interesting solution for hospital benchmarking in general.

### 4.1 Limitations

Data used for model development has been retrospectively collected with known limitations compared to a prospective data assessment. However, it has been stated that data collection mode per se did not influence the discriminatory power of the derived prediction model.^31^ Differences with respect to baseline variables between the patient cohort used for model development and the cohorts the model was applied to may influence the predictive accuracy. However, absolute differences of variable prevalence were acceptable and are unlikely to impact model‐derived predictions relevantly. As administrative data is not stored for research interests but for remuneration reasons, a potential affection of the encoded information is possible. The quality of the results depends to a large extent on the correct encoding of hospital discharge diagnoses.^12^ However, regarding the main discharge diagnosis and the adequacy of hospitalization as well as encoding, there is a continuous evaluation by reimbursement companies/health insurances which supports the assumption of overall valid information. NYHA class assignment is influenced by the subjective assessment of the treating physician, but a potential bias would influence all investigated groups and is likely to be attenuated by the large cohort size. Supporting this information with more objective variables would be desirable, but neither data regarding patients' specific medical history, cardiac imaging, laboratory results, medication nor treatment‐related data was available due to the type and structure of the analyzed database.

## 5 CONCLUSION

Using an ML algorithm processing widely available administrative data, we developed a reliable model to calculate expected in‐hospital mortality rates on a population level in a cohort of inpatients urgently admitted for HF. Applying the model on HF patients’ data during the COVID‐19 pandemic, no significant increase in observed mortality above predicted event rates was found with respect to pandemic phases.

## CONFLICT OF INTERESTS

The authors declare that there are no conflict of interests.

## Supporting information

Supplementary Information

## Data Availability

The data that support the findings of this study are available from the corresponding author upon reasonable request.
