# Supplementary material for: Expected and observed in‐hospital mortality in heart failure patients before and during the COVID‐19 pandemic: Introduction of the machine learning‐based standardized mortality ratio at Helios hospitals
Source: Clin Cardiol. 2021 Dec 23;45(1):75–82. doi: 10.1002/clc.23762 (PMC8799043; doi:10.1002/clc.23762)
Supplement: Supplementary file 1 — Supplementary Information [file CLC-45--s001.docx]

**Supplemental Material**

**Table legends**

Supplemental Table 1: ICD-codes used as tracers for the identification of HF and for the definition of NYHA classes

Supplemental Table 2: ICD-codes used to calculate Elixhauser Comorbidity Score (weighting according to AHRQ algorithm)

Supplemental Table 3: Baseline characteristics within the development dataset overall and as a comparison for training and testing dataset

Supplemental Table 4: Variable importance for the extreme gradient boosting model

**Figure legends**

Supplemental Figure 1: Smoothed curves based on daily hospital admission rates for 2019 and 2020 with non-overlapping 95% CIs defining three different time intervals

Supplemental Table 1

| ICD*-COdes defining heart failure and NYHA classes | |
| --- | --- |
| Heart failure | I11.00; I11.01; I13.00; I13.01; I13.20; I13.21; I25.5; I42.0; I42.6; I42.7; I50.x |
| NYHA class I | I50.11 |
| NYHA class II | I50.12 |
| NYHA class III | I50.13 |
| NYHA class IV | I50.14 |

* International Statistical Classification of Diseases and Related Health Problems (ICD-10-GM [German Modification])

Supplemental Table 2

| ICD*-codes used to calculate Elixhauser comorbidity score | | |
| --- | --- | --- |
| *Item* | *Weight* | *ICD*-10-GM-code* |
| AIDS / HIV | 0 | B20, B21, B22, B23, B24 |
| Alcohol Abuse | -1 | F10, E52, G62.1, I42.6, K29.2, K70.0, K70.3, K70.9, T51, Z50.2, Z71.4, Z72.1 |
| Blood Loss Anemia | -3 | D50.0 |
| Cardiac Arrhythmia | 0 | I44.1, I44.2, I44.3, I45.6, I47, I48, I49, R00.0, R00.1, R00.8, T82.1, Z45.00, Z45.01, Z95.0 |
| Chronic Pulmonary Disease | 3 | I27.8, I27.9, J40, J41, J42, J43, J44, J45, J46, J47, J60, J61, J62, J63, J64, J65, J66, J67, J68.4, J70.1, J70.3 |
| Chronic Renal Failure | 6 | I12.0, I31.1, N18, N19, N25.0, Z49.0, Z49.1, Z49.2, Z94.0, Z99.2 |
| Coagulopathy | 11 | D65, D66, D67, D68, D69.1, D69.3, D69.4, D69.5, D69.6 |
| Congestive Heart Failure | 9 | I09.0, I11.0, I13.0, I13.2, I25.5, I42.0, I42.1, I42.2, I42.5, I42.6, I42.7, I42.8, I42.9, I43, I50 |
| Deficiency Anemia | -2 | D50.8, D50.9, D51, D52, D53 |
| Depression | -5 | F20.4, F31.3 - F31.5, F32, F33, F34.1, F41.2, F43.2 |
| Diabetes Mellitus, Uncomplicated | 0 | E10.0, E10.1, E10.9, E11.0, E11.1, E11.9, E12.0, E12.1, E12.9, E13.0, E13.1, E13.9, E14.0, E14.1, E14.9 (excluding E10.2, E10.3, E10.4, E10.5, E10.6, E10.7, E10.8, E11.2, E11.3, E11.4, E11.5, E11.6, E11.7, E11.8, E12.2, E12.3, E12.4, E12.5, E12.6, E12.7, E12.8, E13.2, E13.3, E13.4, E13.5, E13.6, E13.7, E13.8, E14.2, E14.3, E14.4, E14.5, E14.6, E14.7, E14.8) |
| Diabetes Mellitus, Complicated | -3 | E10.2, E10.3, E10.4, E10.5, E10.6, E10.7, E10.8, E11.2, E11.3, E11.4, E11.5, E11.6, E11.7, E11.8, E12.2, E12.3, E12.4, E12.5, E12.6, E12.7, E12.8, E13.2, E13.3, E13.4, E13.5, E13.6, E13.7, E13.8, E14.2, E14.3, E14.4, E14.5, E14.6, E14.7, E14.8 |
| Drug Abuse | -7 | F11, F12, F13, F14, F15, F16, F18, F19, Z71.5, Z72.2 |
| Fluid And Electrolyte Disorders | 11 | E22.2, E86, E87 |
| Hypertension (combined uncomplicated and complicated) | -1 | I10, I11, I12, I13, I15 |
| Hypothyroidism | 0 | E00, E01, E02, E03, E89.0 |
| Liver Disease | 4 | B18, I85, I86.4, I98.2, K70, K71.1, K71.3, K71.4, K71.5, K71.7, K72, K73, K74, K76.0, K76.2, K76.9, Z94.4 |
| Lymphoma | 6 | C81, C82, C83, C84, C85, C88, C96, C90.0, C90.2 |
| Metastatic Cancer | 14 | C77, C78, C79, C80 |
| Neurological Disorders, other | 5 | G10, G11, G12, G13. G20, G21, G22, G25.4, G25.5, G31.2, G31.8, G31.9, G32, G35, G36, G37, G40, G41, G93.1, G93.4, R47.0, R56 |
| Obesity | -5 | E66 |
| Paralysis | 5 | G04.1, G11.4, G80.1, G80.2, G81, G82, G83.0, G83.1, G83.2, G83.3, G83.4, G83.9 |
| Peptic Ulcer Disease, Excluding Bleeding | 0 | K25.7, K25.9, K26.7, K26.9, K27.7, K27.9, K28.7, K28.9 |
| Peripheral Vascular Disorders | 3 | I70, I71, I73.1, I73.8, I73.9, I77.1, I79.0, I79.2, Z95.81, Z95.88, Z95.9 |
| Psychoses | -5 | F20, F22, F23, F24, F25, F28, F29, F30.2, F31.2, F31.5 |
| Pulmonary Circulation Disorder | 6 | I26, I27, I28.0, I28.8, I28.9 |
| Rheumatoid Arthritis / Collagen Vascular Diseases | 0 | L94.0, L94.1, L94.3, M05, M06, M08, M12.0, M12.3, M30, M31.0, M31.1, M31.2, M31.3, M32, M33, M34, M35, M45, M46.1, M46.8, M46.9 |
| Solid Tumor Without Metastases | 7 | C00, C01, C02, C03, C04, C05, C06, C07, C08, C09, C10, C11, C12, C13, C14, C15, C16, C17, C18, C19, C20, C21, C22, C23, C24, C25, C26, C30, C31, C32, C33, C34, C37, C38, C39, C40, C41, C43, C45, C46, C47, C48, C49, C50, C51, C52, C53, C54, C55, C56, C57, C58, C60, C61, C62, C63, C64, C65, C66, C67, C68, C69, C70, C71, C72, C73, C74, C75, C76, C97 |
| Valvular Heart Disease | 0 | I05, I06, I07, I08, I09.1, I34, I35, I36, I37, I38, I39, Q23.0, Q23.1, Q23.2, Q23.3, Z95.2, Z95.3, Z95.4 |
| Weight Loss | 9 | E40, E41, E42, E43, E44, E45, E46, R63.4, R64 |

* International Statistical Classification of Diseases and Related Health Problems (ICD-10-GM [German Modification])

**Table 3: Baseline characteristics within the development dataset overall and comparing the training and testing dataset**

| Baseline characteristics overall and comparing the training and testing dataset | | | | |
| --- | --- | --- | --- | --- |
| *Variable* | *Total* | *Training dataset* | *Testing dataset* | *P* |
| n | 59,125 | 44,344 | 14,781 | / |
| Age  <65 years [%]  65-74 years [%]  >74 years [%] | 12.6  17.6  69.8 | 12.6  17.5  69.9 | 12.7  17.8  69.5 | 0.949  0.744  0.685 |
| Length of stay  <5 days [%]  5-9 days [%]  >9 days [%] | 37.2  32.8  30.0 | 37.2  32.7  30.0 | 37.0  33.0  30.0 | 0.858  0.861  1.000 |
| Length of ICU stay  0 days [%]  >0 days [%] | 80.3  19.7 | 80.3  19.7 | 80.5  19.5 | 0.882 |
| NYHA class |  |  |  |  |
| NYHA class II [%]  NYHA class III [%]  NYHA class IV [%] | 8.9  42.0  47.4 | 9.0  42.0  47.4 | 8.7  42.1  47.5 | 0.503  0.986  0.921 |
| Gender  female [%]  male [%] | 51.9  48.1 | 51.9  48.1 | 51.8  48.2 | 0.965 |
| Elixhauser comorbidity score | / | / | / | / |
| Cardiac arrhythmias [%] | 62.4 | 62.4 | 62.7 | 0.780 |
| Chronic pulmonary disease [%] | 19.5 | 19.4 | 19.6 | 0.908 |
| Chronic renal failure [%] | 63.0 | 63.0 | 62.9 | 0.980 |
| Deficiency anemia [%] | 5.4 | 5.4 | 5.3 | 0.758 |
| Depression [%] | 5.3 | 5.3 | 5.3 | 0.970 |
| Diabetes, complicated [%] | 22.0 | 22.0 | 22.0 | 0.999 |
| Diabetes, uncomplicated [%] | 18.0 | 18.0 | 18.1 | 0.910 |
| Fluid and electrolyte disorders [%] | 31.3 | 31.3 | 31.3 | 1.000 |
| Hypertension, complicated [%] | 49.6 | 49.6 | 49.7 | 0.987 |
| Hypertension, uncomplicated [%] | 30.1 | 30.2 | 29.8 | 0.574 |
| Hypothyroidism [%] | 13.4 | 13.5 | 13.1 | 0.413 |
| Obesity [%] | 23.3 | 23.4 | 23.0 | 0.645 |
| Peripheral vascular disease [%] | 12.9 | 13.0 | 12.5 | 0.308 |
| Pulmonary circulation disorder [%] | 19.2 | 19.3 | 18.9 | 0.467 |
| Valvular heart disease [%] | 37.7 | 37.8 | 37.3 | 0.605 |
| Weight loss [%] | 6.0 | 5.9 | 6.3 | 0.278 |

^a^ Mean (±standard deviation) presented for continuous variables and percentages for categorical variables

**Supplemental Table 4**

| Variable importance values for the extreme gradient boosting models | | | |
| --- | --- | --- | --- |
| *Variable* | *Elixhauser comorbidities* | *Elixhauser Score* | *Elixhauser Index* |
| Age | 46.3 | 37.0 | 37.3 |
| Length of stay | 100.0 | 100.0 | 100.0 |
| Length of ICU stay | 59.3 | 52.3 | 52.1 |
| NYHA class II | 7.6 | 6.6 | 6.1 |
| NYHA class III | 12.9 | 10.8 | 10.3 |
| NYHA class IV | 62.0 | 56.7 | 56.0 |
| Gender | 2.3 | 0.9 | 0.4 |
| Admission year | 2.4 | 0.0 | 0.0 |
| Elixhauser comorbidity score | / | 48.4 | / |
| Elixhauser comorbidity index | / | / | 32.6 |
| Cardiac arrhythmias [%] | 1.1 | / | / |
| Chronic pulmonary disease [%] | 1.3 | / | / |
| Chronic renal failure [%] | 0.6 | / | / |
| Deficiency anemia [%] | 0.2 | / | / |
| Depression [%] | 0.0 | / | / |
| Diabetes, complicated [%] | 0.4 | / | / |
| Diabetes, uncomplicated [%] | 0.2 | / | / |
| Fluid and electrolyte disorders [%] | 21.9 | / | / |
| Hypertension, complicated [%] | 9.2 | / | / |
| Hypertension, uncomplicated [%] | 4.3 | / | / |
| Hypothyroidism [%] | 0.4 | / | / |
| Obesity [%] | 1.2 | / | / |
| Peripheral vascular disease [%] | 0.6 | / | / |
| Pulmonary circulation disorder [%] | 1.1 | / | / |
| Valvular heart disease [%] | 1.8 | / | / |
| Weight loss [%] | 8.8 | / | / |

**Supplemental Figure 1: Smoothed curves based on daily hospital admission rates for 2019 and 2020 with non-overlapping 95% CIs defining three different time intervals**


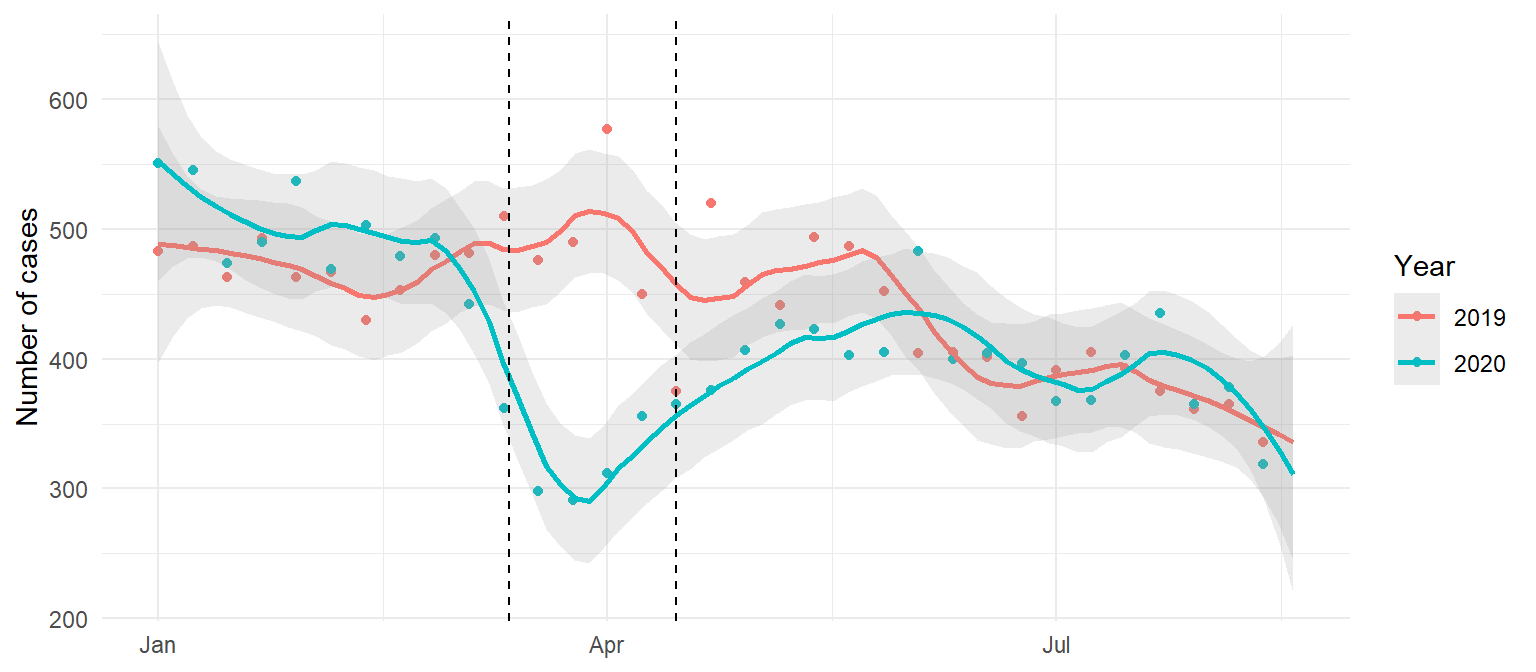


Gray areas represent 95% CIs. Dashed vertical bars represent begin of the deficit (first bar) and the resumption (second bar) period.
